# Emotion Recognition from Physiological Channels Using Graph Neural Network

**DOI:** 10.3390/s22082980

**Published:** 2022-04-13

**Authors:** Tomasz Wierciński, Mateusz Rock, Robert Zwierzycki, Teresa Zawadzka, Michał Zawadzki

**Affiliations:** 1 Faculty of Electronics, Telecommunications and Informatics and Digital Technologies Center, Gdańsk University of Technology, 80-233 Gdańsk, Poland; tegra@eti.pg.edu.pl; 2 Faculty of Electronics, Telecommunications and Informatics, Gdańsk University of Technology, 80-233 Gdańsk, Poland; mateuszrockm@gmail.com (M.R.); robert.zwierzycki@gmail.com (R.Z.); 3 Independent Researcher, 80-233 Gdańsk, Poland; michal.j.zawadzki@gmail.com

**Keywords:** affective computing, emotion recognition, multi-modality, graph neural network, biosignals, biosensors, bioelectrical signals

## Abstract

In recent years, a number of new research papers have emerged on the application of neural networks in affective computing. One of the newest trends observed is the utilization of graph neural networks (GNNs) to recognize emotions. The study presented in the paper follows this trend. Within the work, GraphSleepNet (a GNN for classifying the stages of sleep) was adjusted for emotion recognition and validated for this purpose. The key assumption of the validation was to analyze its correctness for the Circumplex model to further analyze the solution for emotion recognition in the Ekman modal. The novelty of this research is not only the utilization of a GNN network with GraphSleepNet architecture for emotion recognition, but also the analysis of the potential of emotion recognition based on differential entropy features in the Ekman model with a neutral state and a special focus on continuous emotion recognition during the performance of an activity The GNN was validated against the AMIGOS dataset. The research shows how the use of various modalities influences the correctness of the recognition of basic emotions and the neutral state. Moreover, the correctness of the recognition of basic emotions is validated for two configurations of the GNN. The results show numerous interesting observations for Ekman’s model while the accuracy of the Circumplex model is similar to the baseline methods.

## 1. Introduction

Automatic emotion recognition is an interdisciplinary research field that deals with the algorithmic detection of human affect from a variety of sources [1]. In recent years, much research, motivated by the success of the graph neural network (GNN) model in graph data, has focused on emotion recognition using GNNs. The GNN is an approach that extends deep-learning techniques with the ability to operate on data represented as a graph and not as Euclidean data. In emotion recognition, it is primarily performed on (but not limited to) EEG (electroencephalography) biosignals, where the relationships between EEG channels have an influence on the recognized emotions [2]. This is similar to another challenge in the utilization of EEG biosignals: classifying the sleep stage. The research described in [3] introduces the GraphSleepNet—a graph convolutional network (GCN)—for this purpose. The authors of this paper perceived the GraphSleepNet features to be a premise that an analogous network can be used for emotion recognition. The GraphSleepNet features are as follows:

- It combines spatial–temporal convolution and spatial–temporal attention mechanisms;
- It represents the pairwise relationship between nodes to dynamically construct an adjacency matrix;
- It is easy to expand the model by other types of biosignals.

Emotions can be recognized in various models, with two of the most popular ones being Ekman’s model describing six basic emotions (happiness, anger, sadness, surprise, fear, and disgust) [4], often extended with the neutral state, and the Circumplex model [5], where emotions are represented as two-dimensional vectors of valence (unpleasant/pleasant) and arousal (deactivation/activation). In the course of this research, the GraphSleepNet neural network was adjusted to emotion recognition for Ekman’s model and the Circumplex model, then named the GraphEmotionNet.

One of the motivations of the work was to study the aptitude of applying the architecture of the GNN network utilized in GraphSleepNet for emotion recognition. No less important a motivation, but less technical, was the aim to study the potential of the architecture and applied differential entropy (DE) features in recognizing the six basic emotions and the neutral state continuously while performing activities. This was particularly important, as research often utilizes DE features for recognizing emotions, but mainly in the Circumplex model [6,7] or for three emotional states (positive, neutral and negative) [7,8,9,10,11] and, to the best of our knowledge, none apply the Ekman model. Moreover, because a person can feel and express more compound emotions, we wanted to apply a solution which does not return only one of the basic emotions or the neutral state, but an emotion state vector defined as a set of values from a specific range assigned to the emotional states [12].

The goal of the research is to validate GraphEmotionNet for automatic emotion recognition. It is assumed that the goal of the research is fulfilled when the answers to the following research questions are found:

1. RQ1—What is the accuracy of the valence and arousal predicted by GraphEmotionNet?
2. RQ2—What is the correctness of the emotion state vectors for Ekman’s model with the neutral state predicted by GraphEmotionNet for a single moment of time?
3. RQ3—In what way do the modalities used in GraphEmotionNet and the emotion recognized influence the answers of RQ2?

**Figure 1:**
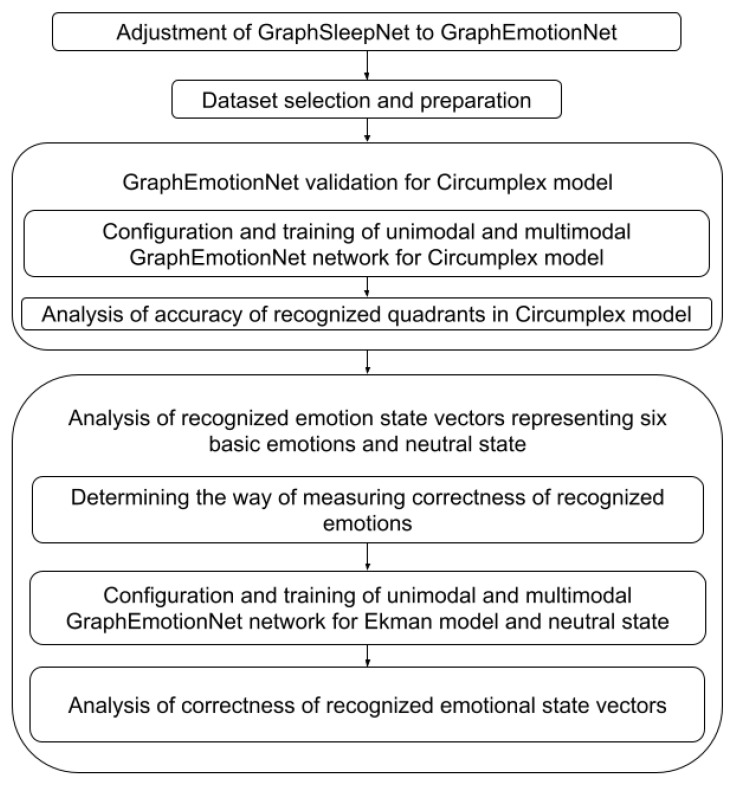
Research methodology.

To find the answers to these questions, the research methodology depicted in Figure 1 was applied. The following tasks were designed:

1. Transformation of the GraphSleepNet GNN to GraphEmotionNet and then further preparation of the GNN to work in two configurations utilizing the unimodal and multimodal approaches.
2. Selection of a dataset that provides continuous annotations, as much as possible, and obtaining emotions treated as ground truth continuously while performing the activity.
3. GraphEmotionNet validation for the Circumplex model to prove its applicability for emotion recognition. The aim of this task was not to improve the accuracy in relation to the baseline methods, but to show a similar accuracy to other research. Within this task, two substasks were performed:
  - Configuration and training of GraphEmotionNet for the Circumplex model for the unimodal approach (EEG only) and multimodal approach (EEG with at least two other biosignals).
  - Analysis of the accuracy of the recognized quadrants in the Circumplex model, where the quadrants are understood in terms of high or low values of valence and arousal. This analysis was performed with respect to baseline methods.
4. Analysis of the recognized emotion state vectors representing the six basic emotions and the neutral state. The aim of this task was to check the correctness of the recognized emotion state vectors and provide the main contribution of this paper: results showing which basic emotions can be recognized with the highest correctness utilizing DE features in GraphEmotionNet and in what way the used modalities influence this. Within this task, three subtasks were performed:
  - Determination of the method for measuring the correctness of the recognized emotions, which was achieved by choosing the similarity measure, allowing for the comparison of the two emotion state vectors.
  - Configuration and training of GraphEmotionNet for the Ekman model with a neutral state for the unimodal approach (EEG only) and multimodal approach (EEG with at least two other biosignals).
  - Analysis of the chosen similarity measure of the recognized emotion state vectors, which made it possible to draw some conclusions. This analysis was not performed with respect to baseline methods, as to the best of our knowledge no similar analysis has been published.

The paper is organized as follows. Section 2 describes recent trends in automatic emotion recognition with a special focus on the application of graph neural networks. The description of GraphSleepNet and the steps performed to build GraphEmotionNet can be found in Section 3. In Section 4, the design of the experiments to analyze the accuracy of the recognized quadrants in the Circumplex model and to analyze the recognized emotional state vectors is described. The results of the performed analysis are presented in Section 5. Finally, the results are discussed in Section 6.

## 2. Related Work

To familiarize the readers with the topic of automatic emotion recognition, two main aspects are presented. Firstly, Section 2.1 shows recent trends in automatic emotion recognition from physiological channels. Secondly, the approaches taken in the usage of graph neural networks to recognize emotions are presented in Section 2.2.

### 2.1. Automated Emotion Recognition from Physiological Channels

Following [13], automatic emotion recognition is a collection of methods that enables the processing and classification of various signals to detect a given emotion from a set of available emotions. The aim of studies focusing on automatic emotion recognition is always the same, however they differ in terms of recognized emotions, observation channels used, utilized techniques, used dataset, and applied unimodal or multimodal approach [14,15,16].

#### 2.1.1. Models for Representing Emotions

Following [17], there are three major model types for representing emotions: discrete, dimensional and componential [18]. Discrete models distinguish a set of basic emotions and describe each affective state as belonging to a certain emotion from the predefined set. One of the best-known and extensively adapted discrete representation models is Ekman’s six basic emotions model, which includes happiness, anger, disgust, surprise, sadness, and fear [4]. Dimensional models represent an emotional state as a point in a multi-dimensional space and the most adapted model uses continuous valence and arousal dimensions, which are also frequently extended with an additional dimension of dominance [5]. Componential models use several factors that constitute or influence the resulting emotional state and the most adopted OCC model defines a hierarchy of emotion types representing all the possible states that might be experienced [19]. The analysis of the emotion recognition solutions reveals that there is no single commonly accepted standard model for emotion representation. The continuously adapted Ekman’s six basic emotions and valence-arousal (also called Circumplex) models are widely used in emotion recognition solutions [18]. Recently, more papers have been discussing the nature and modeling of emotions in affective computing [20,21], but most of the research is still focused on dichotomous models of emotions [22].

#### 2.1.2. Observation Channels

During the emotion recognition process, various life activities (conscious and unconscious actions of a human body, which generate specific symptoms of an emotional state) [23] are analyzed. To the most-analyzed life activities belong: various types of movement, a sound made by the person, physiological activities such as heart and brain activity, unconscious muscle activity, respiration, and perspiration or thermal regulation. The life activities might be recorded via observation channels, which are mediums for the registration of a signal holding information on observable symptoms. The observation channel refers to a type of signal obtained rather than a physical medium. Analysis of psychological observation channels is an alternative to movement and sound analysis. People control movements and sounds so it is easy to manipulate them [24,25]. This analysis is also suitable for those who are not able to express emotions in other ways. The most widely used physiological observation channels in emotion recognition include EEG (electroencephalography), ECG (electrocardiography), and GSR (galvanic skin response, often called EDA-electrodermal activity in recent works), but are not limited to these alone (e.g., there is also temperature, chest size, EMG-electromyography, and BVP-blood volume pulse).

The EEG observation channel is used in emotion recognition because it reflects in some way a person’s thoughts [26,27]. Therefore, EEG is widely used in emotion recognition as an observation channel of brain activity and the studies focused on this channel are described in recent surveys [24,28,29]. Knowing that the heart is connected with the brain via the autonomic nervous system (ANS), emotional experience causes some changes in heart rhythm, which can be detected through ECG readings [25]. The current research on automatic emotion recognition focused on ECGs are reviewed in [25,30,31]. The other widely used channel for emotion recognition is GSR, which is mainly a result of two things. Firstly, research [32] has shown that GSR is a reliable identifier of physiological arousal, and secondly, devices that measure GSR are more widely available [33] and wearable technologies provide more opportunities to measure GSR in real-world contexts [34,35]. The recent research reviews can be found in [35,36,37].

#### 2.1.3. Techniques and Approaches for Multimodal Processing

Various techniques for automatic emotion recognition are used. The classification introduced in [24] introduces Deep Machine Learning-based systems and Shallow Machine Learning-based systems. Deep learning-based systems, among others, include CNN (Convolutional Neural Network), DNN (Deep Neural Network), RNN (Recurrent Neural Network), and GNN. On the other hand, shallow learning-based systems include SVM (Support Vector Machine), kNN (k Nearest Neighbor), DT (Decision Tree), etc. The main difference between the shallow and deep learning methods is the different features utilized. Shallow learning is based on predefined mathematical features. On the contrary, the deep learning models derive their own features (directly from the data) to create the best model.

Two main approaches to multimodal emotion recognition can be distinguished: early fusion and late fusion [38]. Early fusion is a process of combining features from diverse types of modalities for further analyses using statistical or machine learning techniques. This makes it possible to find interrelationships in modalities at the initial phase. On the other hand, late fusion focuses on the processing of each modality separately using different models for different modalities. After that, a new emotion is determined using different machine learning techniques using previously recognized emotions. Moreover, there is also a hybrid version of these approaches [39]. In this approach, some features can be combined as an input for a machine learning model (early fusion), after which late fusion is applied.

#### 2.1.4. Datasets

The comprehensive review of affective datasets [40] published in 2019 lists five multimodal datasets that contain bioelectrical signals of interest.

To familiarize the reader with the data, a basic comparison of the most popular affective datasets has been prepared in Table 1 and Table 2. Table 1 lists other works regarding emotion recognition that made use of said datasets as training data along with the predicted labels. Table 2 lists the bioelectrical signals found in each dataset along with the number of participants and a short description of the stimuli used to elicit emotional reactions.

### 2.2. Graph Neural Networks for Automatic Emotion Recognition

A graph neural network is a method of deep learning used for graph data structures [43]. Given the fact that presently, more and more works use data represented as graphs instead of Euclidean data, this provides an opportunity to use the unique properties of graph neural networks not only in the field of emotion recognition with the usage of bioelectrical sensors [2], but also in the domain of facial expression detection (facial emotion recognition) [44], emotion context detection in dialogue/conversation between humans [45,46], and emotion recognition from speech [47] with the use of a skeleton model to recognize emotions on the basis of gestures/body layout [48] or a fusion of two or more emotion detection methods, e.g., from visual and audio inputs [49,50].

Besides the application of graph neural networks in emotion recognition, there are other various applications, such as analyzing the structures of a given graph (graph mining) [51], recognizing relationships between words in text classification [52], and drawing relations between objects in image classification [53]. One of the advantages of graph neural networks is the assumption that data instances are related to each other by specific relationships [54]. This unique property enables us to operate with a new type of data: data that can have relations between each other, for example bioelectrical signals, social interactions, natural language processing, etc. In this study, the focus has been set on the use of graph neural networks in the field of affective computing, or to be more precise, to automatically recognize emotions from various bioelectrical signals such as EEG, ECG, and GSR.

The common approaches to using GNNs for emotion recognition differ by the used modalities, methods of graph construction and, in the case of multimodal solutions, the approach to data fusion. The method of graph construction can be classed as either static or dynamic. A statically constructed graph has edge weights determined by features of the data that remain unchanging during the training process.

Statically constructed graphs are based on a priori knowledge of the relations between the nodes and most commonly express distances between specified points in physical space, such as relations between points of the human body. These can be used to summarize video footage of a persons gait by creating a spatial–temporal graph based on joint positioning, or footage of the face by assigning graph nodes to specific features of the face and expressing distances between them using edges [48,55,56,57]. Included in this category are also the positions of sensors used to collect data, such as EEG electrodes [58,59,60], or even calculated values expressing relations between segments of data [61,62]. Any models that are fed a ready adjacency matrix as opposed to the adjacency matrix being a product of the network are considered to be static.

Dynamically constructed graphs are graphs whose adjacency matrix constructions are learned during model training and dynamically changed to maximize specific metrics [3,63,64,65]. These methods make no prior assumptions about the existing relations between the nodes and instead learn them based on the training data. These are most commonly used with multichannel unimodal data—such as EEG—which allows for the easy definition of graph nodes and the extraction of common features that can be used for training the graph edges.

In terms of modality, the training data can be either unimodal or multimodal, and for the multimodal approach some kind of fusion is applied. In the work of [57], the integration of multimodal data from videos, audio, and text was used as a late fusion model for emotion prediction into four classes (happy, sad, angry, and neutral). Ref. [15] uses late fusion with modalities for the emotion recognition task. The research described in [66] also uses late fusion of the gate and EEG modalities, where the use of a graph neural network was applied for feature extraction of the skeleton frames (gait). The solution presented in [67] utilizes early fusion of modalities for multimodal emotion recognition, where a graph neural network is used to model the correlation between neurons and emotion recognition.

## 3. Graph Neural Network

This section describes the performed piece of works related to the task involving the adjustment of GraphSleepNet to GraphEmotionNet and the two concerning the configuration and training of the unimodal and multimodal GraphEmotionNet network for both models. The graph neural network model used is based heavily on the GraphSleepNet model [3], which is used to classify sleep stages with the use of both spatial–temporal convolution and spatial–temporal attention mechanisms. This model was chosen due to its use of EEG data and high classification accuracy (88.90%). For the purposes of this study, the model was adapted to classify emotions.

### 3.1. GraphSleepNet Model

The GraphSleepNet model is an adaptive Spatial-Temporal Graph Convolutional Network (ST-GCN) used on electroencephalogram (EEG) data for sleep stage classification. It extracts the intrinsic connections of the EEG channels, creating an adjacency matrix. Instead of using a predefined connection structure, the graph edges between individual signal channels are learned adaptively. This means that the graph structure is created dynamically during the learning process. Moreover, it successfully captures both the spatial features of the signal and the timed changes of the signal using spatial and temporal convolutions, respectively. The spatial features are determined from each sleep stage network by aggregating information from neighbor nodes. In contrast, the temporal dependencies from the neighbor sleep stages are used to extract the temporal features.

The overall structure of the GraphSleepNet model remains unchanged for the purposes of emotion classification. The adaptive graph learning layer produces the adjacency matrix from the input data by minimizing the graph learning loss function. The loss function was designed to generate larger edge weights between nodes represented by feature vectors that are close together in terms of euclidean distance. It also contains a term that grows along with the total weights to avoid creating a fully connected graph. To avoid the trivial solution of setting all of the weights (and consecutively, all of the edges) to zero, a cross entropy loss is also added.

A detailed description of the GraphSleepNet architecture is provided in the paper [3].

### 3.2. Adaptation of the GraphSleepNet to Recognize Emotions—GraphEmotionNet

**Figure 2:**
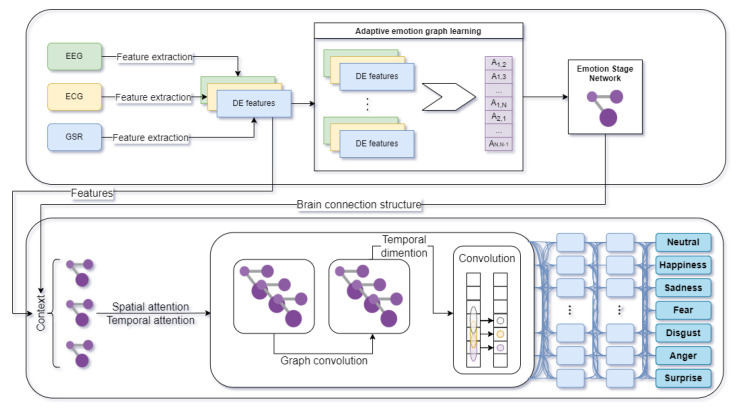
Overall architecture of GraphEmotionNet (own elaboration based on GraphSleepNet [3]).

To conduct the experiments, a modified version of the aforementioned GraphSleepNet model was used. The GraphEmotionNet overall architecture is shown in Figure 2. Firstly, the differential entropy (DE) features are extracted from EEG or a combination of EEG, ECG, and GSR biosignals. Then comes the adaptive emotion graph learning process. Next, the combination of extracted DE features and brain connection structure serves as an input to the Spatial–Temporal Graph Convolution. The final stage is emotion classification to the Ekman model, with an additional neutral stage as shown in Figure 2, or to the Circumplex model. The results from the network are given as a vector of the probabilities of belonging to the emotion classes.

The modified network takes only the extracted DE features [68] from the EEG signal or from the combination of EEG, ECG, and GSR signals as an input. The variant of the network that processes all three modalities does so by constructing a single 17-node graph that combines all of the channels. As stated in [69], DE has the property of measuring the average information of a random variable which is capable of separating EEG signals from low and high frequency energy.

The output layer returns the emotion classes from the selected model depending on the experiment’s assumptions. Both the six classes of emotions specified in Ekman’s model extended with the neutral emotion, and the quadrants in the Circumplex model (high valence high arousal (HVHA), low valence high arousal (LVHA), high valence low arousal (HVLA), and low valence low arousal (LVLA), were used.

To avoid overfitting, the input data was divided into training and validation sets using a 49-fold cross-validation strategy. Each fold in the dataset consisted of data from 11 viewing sessions (as defined in the AMIGOS dataset as a participant viewing a single video). The viewing sessions were of different lengths and therefore resulted in a different number of samples per fold. The training was therefore performed 49 times with the final reported results being the total of all the validation folds from their respective training sessions. The case of underfitting was addressed by training the data over 50 epochs, and the callback mechanism was used to obtain the model with the highest accuracy score. The used hyperparameters are presented in Table 3.

In the case of predicting emotions to the appropriate quadrant in the Circumplex model, the performance of the prepared GNN was evaluated with 10-fold cross-validation. The training and test set was randomly chosen by subject-independent schemes. The used hyperparameters are presented in Table 4.

## 4. Experiments Design

According to the designed research methodology depicted in Figure 1, performing the experiments demands first Dataset selection and Preparation, which is described in Section 4.1 and Section 4.2. The tasks Graph EmotionNet validation for Circumplex model and Analysis of recognized emotion state vectors representing six basic emotions and neutral state also require the configuration and training of the GraphEmotionNet. These issues were described in Section 3. Thus, the analysis design is presented in Section 5.3. Section 5.1 first presents the design steps for Determining the method of measuring correctness of recognized emotions and secondly the design of the Analysis of correctness of recognized emotional state vectors.

### 4.1. Dataset

Following [70], the AMIGOS dataset is designed for research on affective reactions based on neurophysiological signals and video recordings of the face and whole body. The collected data comes from experiments in which participants individually or in small groups watched short and long movies that evoke strong emotions. This makes it possible to analyze the video duration and the impact of social context on the participants’ emotional state. Moreover, the dataset provides information about personality and mood for each of them.

The collected data in the dataset consists of participant profiles, neurophysiological signals, participant videos, and emotional state assessments.

AMIGOS provides three types of neurophysiological signals:

1. Electroencephalogram (EEG) that contained fourteen channels (AF3, F7, F3, FC5, T7, P7, O1, O2, P8, T8, FC6, F4, F8, AF4);
2. Electrocardiogram (ECG) that contained two channels;
3. Galvanic skin response (GSR) with only one channel.

The signals are available in the originally received form, and in a preprocessed and segmented form.

The participants were recorded using a HD frontal camera pointed at the face and a second camera capturing RGB and depth videos covering the whole body. All video recordings were precisely synchronized with the neurophysiological signals.

The emotional state assessments were obtained using two methods:

1. The self method was obtained from the participants’ self-assessment made by completing special questionnaires about their emotional state at the beginning of each experiment and about their emotional state, video liking, and familiarity at the end of each experiment.
2. The external method was based on ratings of the arousal and valence levels made by three independent annotators every 20 s using the participants’ face recordings.

The data from the scenario of the short videos experiment was chosen for the designed experiments. It involved 40 participants, where each individually watched a set of short recordings with a duration not exceeding 250 s. These recordings were taken from feature-length films and were selected to evoke specific affective states. Each was classified into one of four categories: HVHA, HVLA, LVHA, and LVLA, referring to the quadrants of the two-dimensional model of emotion representation (where the letters V and A stand for valence and arousal, while H and L indicate high and low levels of the given feature). The list of movies used in the study included 16 positions, four for each category. The order in which they were viewed differed for each participant [71].

The AMIGOS dataset was chosen from the set of multimodal datasets mentioned in Section 2 due to the fact that it contains all of the necessary modalities that are essential to conduct multimodal experiments, and it is a relatively good quality dataset with preprocessed data. The DEAP dataset was not chosen because it is a relatively old dataset (2012). The SEED dataset contains data collected only from the EEG modality, so this dataset would be insufficient for the experiments conducted in this article. The ASCERTAIN dataset contains all of the necessary modalities (EEG, ECG, GSR), so theoretically could be used in this article. However, it does not contain preprocessed data like the AMIGOS dataset. Furthermore, the EEG modality was obtained at a frequency 32 Hz, whereas the AMIGOS dataset contained EEG data obtained with a sampling rate of 128 Hz, which is the minimum frequency for obtaining the range of all wave types from an EEG, based on the given articles [72,73]. Another advantage of the AMIGOS dataset was the continuous annotations made by three external annotators in 20-s time intervals. To the best of our knowledge, only the AMIGOS dataset from the above-mentioned datasets contains continuous external annotations in the given time period. In summary, the decision to use the AMIGOS dataset was made due to its higher sampling frequency and availability of preprocessed multimodal bioelectrical data.

### 4.2. Preprocessing

The analysis of the correctness of the recognized emotional state vectors requires the training and test set to be prepared as a time series of vectors of Ekman’s emotions with the neutral state. The AMIGOS dataset provides two types of annotations—self and external. Only self-annotations provide Ekman’s emotions. However, self-annotations are associated with the whole video, and there is no information on how the emotions changed over time while watching. Thus, the frontal face videos were processed by the Noldus Face Reader software, which is software developed by Nodulus to recognize emotions from videos of faces. It analyzes facial expressions to determine the emotional state of a person on a recording. As a result, the emotion recognition solution returns a vector of the classified emotions in up to six categories according to the Ekman model (happiness, sadness, fear, disgust, anger and surprise) with the additional neutral emotion. Additionally, Face Reader provides estimated valence and arousal. All Ekman’s emotions, neutral state, as well as valence and arousal, have 1 s sampling rates and take values between 0 and 1 (the only exception is valence, where the value range is <−1, 1>). Face Reader also provides information about the detected Ekman’s emotions with the neutral state and times when these emotions are detected.

Consistency between the recognized emotions and annotations were achieved. In the first step, the consistency analysis between the self-annotations and Ekman’s emotions with the neutral state predicted by Face Reader was performed. To do this, the information from Face Reader about the detected emotions was used. Exemplary information about the detected emotions by Face Reader for the first participant watching the 80th movie is presented in Listing 1.

**Listing 1.** Emotions recognized by Face Reader for the first participant watching the 80th movie.Video TimeEmotion00:00:00.000Unknown00:00:01.083Neutral00:00:03.750Unknown00:00:07.833Neutral00:00:09.500Disgusted00:00:14.416Neutral00:00:16.958Disgusted00:00:17.541Surprised00:00:20.000Neutral00:00:29.166Surprised00:00:31.000Neutral00:00:35.958Disgusted00:00:36.791Surprised00:00:41.166Neutral00:01:31.625Happy00:01:32.875Neutral00:01:42.791END

For each participant and each video, a binary matrix $m_{1}$ of detected emotions was generated with one set when the specific emotion was detected while watching the specific video, and 0 otherwise. In Listing 1, it is seen that disgust, surprise, happiness, and the neutral state were recognized, resulting in the $m_{1}$ matrix row depicted in Table 5. This matrix was compared with the analogical binary matrix $m_{2}$ of self-annotations. The row representing the annotated emotions corresponding to those depicted in Table 5 is presented in Table 6 (only anger was annotated).

The consistency measure was calculated for each emotion separately and expresses for each specific emotional state the percentage of correctly detected 1 s, i.e., the number of 1 s in the column representing the particular emotion in the $m_{1}\;AND$ $m_{2}$ (a logical AND is performed for the values of the corresponding cells in both matrices) matrix out of all annotated 1 s (i.e., the 1 s in the analogical column of $m_{2}$). This consistency is low for all emotions except for the neutral state. For the neutral state it is equal to 93%, for happiness 14%, for disgust and sadness 7%, for surprise 4%, for fear 3%, and for anger 2%.

**Figure 3:**
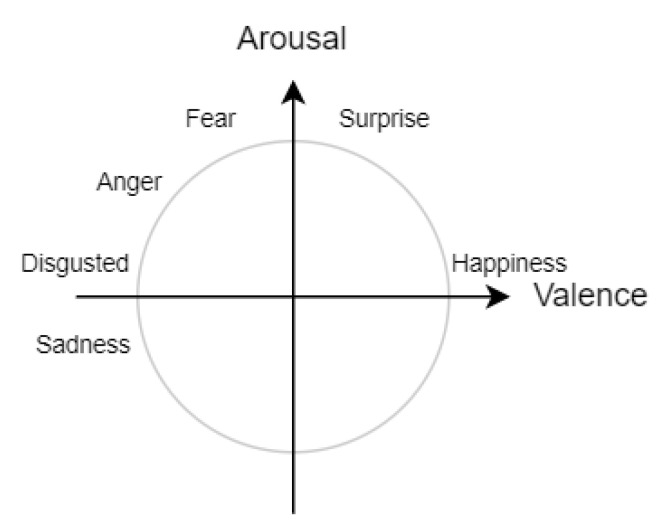
Ekman’s emotions on Circumplex model (own elaboration based on [81]).

Such low consistency of recognized emotions resulted in an analysis of the internal consistency of the self-annotations being performed. This analysis checks the consistency between the self-annotated values of valence and arousal with the self-annotated Ekman’s emotions. In this analysis, the mappings of Ekman’s emotions to the Circumplex model are used. Happiness is in the HVHA quadrant; sadness in LVLA; fear, anger and disgust in LVHA, and surprise when the valence is in the middle of the range of the values and arousal is high, as presented in Figure 3. This rule was used for the analysis of the self-annotations with a deviation of 10% on the plus and minus from the middle of the range for valence when surprise is analyzed. For each annotated valence, arousal and Ekman’s emotions, it is checked whether any of the possible Ekman’s emotions with respect to the valence and arousal values are annotated (if yes, the annotation is treated as consistent). In Table 7, an example of checking the internal consistency of self-annotations is presented. After watching the nineteenth movie, the first participant annotated valence to 1 and arousal to 6.46 (the range of values is <0, 10>). This correlates with the LVHA quadrant. In this quadrant, there are three Ekman’s emotions: fear, anger, and disgust. The annotated external emotions were disgust and fear. According to the rule, if at least one emotion from those possible is annotated, the annotation is treated as consistent, and the case is determined as consistent.

The percentage of consistent annotations was 17.8%. These results confirm that post-stimuli self reports can be imprecise, e.g., due to the time-varying nature of human emotions [74,75,76].

In consequence, an analysis of the consistency between the external-annotations and the recognized valence and arousal by Face Reader was performed. In AMIGOS, external annotations are made by three experts every 20 s. Thus, for each timestamp the valence and arousal were averaged and mapped to 1 s samples. These values were compared with those predicted by Face Reader. The consistency of valence and arousal were analyzed separately as well as the consistency of the obtained quadrants. If the external annotations and estimates obtained from Face Reader for valence/arousal belonged to the same class (high or low) then the sample was treated as consistent. The consistency for valence was 72%, and for arousal was 78%. When the recognized and annotated quadrants were compared, the consistency was equal to 63%. These values reflect typical accuracy for the AMIGOS dataset [77,78,79,80] which allowed us to use Ekman’s emotions obtained from Face Reader in the experiments.

The raw bioelectrical signals divided into one second epochs were processed to extract DE features for each channel of EEG, ECG, and GSR from nine crossed frequency bands: 0.5–4 Hz, 2–6 Hz, 4–8 Hz, 6– 11 Hz, 8–14 Hz, 11–22 Hz, 14–31 Hz, 22–40 Hz, and 31–50 Hz. In the case of the ECG signal, the DE features were extracted from its NN interval—the intervals between consecutive heartbeat detections— and converted to the 128 Hz sampling rate using cubic interpolation. The processed biosignal data were used as an input into GraphEmotionNet in both analyses. Additionally, in the analysis of the accuracy of the recognized quadrants in the Circumplex model, a 50% overlap was applied during the division into epochs that were split into 2 s epochs to improve the model training. The DE values can be highly responsive to the length of the signal and applying an overlap makes it possible to verify different lengths of segments without reducing the sample size. The improvement was within the experimental error and therefore was not reproduced for Ekman labels. Applying an overlap for the Ekman labels corresponding to 1-s-long signal segments would additionally require the use of interpolation methods.

#### Analysis of the Accuracy of Recognized Quadrants in the Circumplex Model

Within this analysis, GraphEmotionNet provides the probabilities of classification to the specific quadrant. The one with the highest probability is chosen. The aim of the analysis is to calculate the accuracy metric of this classification and compare them with baseline methods to show the applicability of GraphEmotionNet for emotion recognition. The two experiments were designed as depicted in Table 8. The first experiment applies the unimodal approach and the second applies the multimodal approach. In Experiment 1, the model takes the extracted DE features for the EEG signal. When the multimodal approach is applied in Experiment 2, the model takes the extracted DE features for the combination of the EEG, ECG, and GSR bioelectrical signals.

The ground truth data for these experiments, analogically as for the analysis of the consistency between the external annotations and the recognized valence and arousal by Face Reader, are derived from the external annotations. The values of the external annotations were averaged (average value calculated from the annotations made by the three experts). The average value was mapped to 1-second samples and for each sample the quadrant in the Circumplex model was determined.

### 4.3. Analysis of Recognized Emotion State Vectors Representing Six Basic Emotions and Neutral State

#### 4.3.1. Determining the Method of Measuring Correctness of Recognized Emotions

The emotion state vector is recognized by the GNN in each moment of time. Its correctness is checked by calculating the cosine similarity for each sample. The cosine similarity was chosen as a similarity measure taking into account both the values assigned to the specific points as well as the direction of the resultant vector, which is especially important when small values are recognized for the compared vectors and the Euclidean distance may vary slightly.

Two approaches for cosine similarity analysis are applied. Firstly, the averaging of the cosine similarity is analyzed. Secondly, the thresholds that make it possible to categorize samples as consistent, semi-consistent, or inconsistent are determined, and then the sizes of particular classes are further analyzed. To determine the thresholds the following steps are performed:

1. A set of about 100 pairs of emotion state vectors is obtained (one vector is recognized by Face Reader and the other one is generated by GraphEmotionNet). The pairs are chosen in such a way that for each dominant emotion (the one with the highest value representing the probability assigned), the number of pairs is the same.
2. Five persons, not knowing the value of the cosine similarity, independently annotate each pair using the values 0, 1, and 2 to denote the pair as non-similar, semi-similar, and similar, respectively.
3. The class of the pair is determined based on the average value of the annotated values; if the average value is ≥1.5, the pair is found to be consistent, if the average value is in the range <0.5, 1.5), the sample is found to be semi-consistent, and if the average value is <0.5, the sample is found to be inconsistent.
4. The tresholds are determined based on the distribution of values of cosine similarity within each class and the minimum value of cosine similarity within these classes.

#### 4.3.2. Analysis of Correctness of Recognized Emotional State Vectors

As depicted in Table 9, the four experiments (3–6) predicting the emotions in Ekman’s model with the additional neutral emotion were designed. Two of these experiments applied the unimodal approach (Exp. 3 and Exp. 5), and the other two applied the multimodal approach (Exp. 4 and Exp. 6). Analogically as for the Circumplex model, in the experiments with one modality, the model takes the extracted DE features for the EEG signal. When the multimodal approach is applied, the model takes the extracted DE features for the combination of EEG, ECG, and GSR bioelectrical signals.

**Figure 4:**
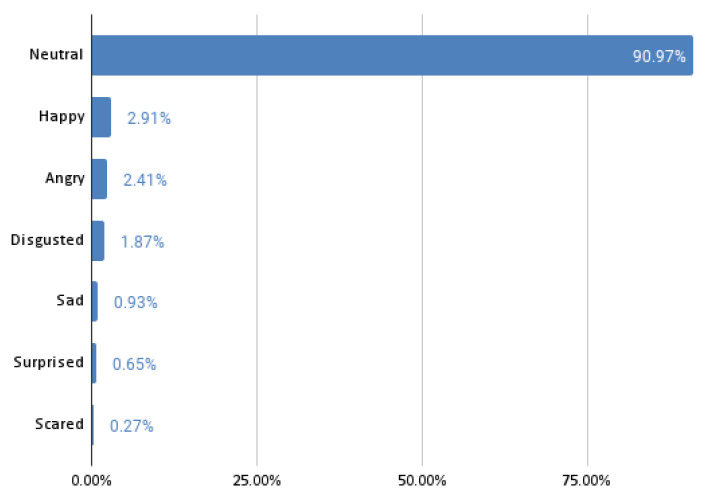
Percentage of samples for the specific dominant emotions.

In this analysis, the ground truth data are the emotion state vectors obtained from Face Reader. The experiments return the probability distribution of the six Ekman’s emotions and the neutral state. In the 5th and 6th experiments, the same model architecture, input, and target data are used as in the 3rd and 4th experiments. However, the training of the model is changed to use class weights that make the model eight times more sensitive to emotions other than neutral. The reason for changing the sensitivity is the vast number of samples in which the neutral state is a dominant emotion. The percentage of samples for the specific dominant emotions are depicted in Figure 4.

## 5. Experiments Results

The results of the experiments are separately described for the analysis of accuracy of the recognized quadrants in the Circumplex model in Section 5.1 and the analysis of the recognized emotion state vectors representing the six basic emotions and neutral state in Section 5.3.

### 5.1. Analysis of the Accuracy of the Recognized Quadrants in the Circumplex Model

The results of the accuracy analysis are presented first in the form of confusion matrices for both experiments, and second in comparison to other baseline methods. It is worth remembering that the aim of this analysis is to prove the applicability of GraphEmotionNet for emotion recognition in order to utilize it in the next step for the analysis of the recognized emotion state vectors representing the six basic emotions and the neutral state.

**Figure 5:**
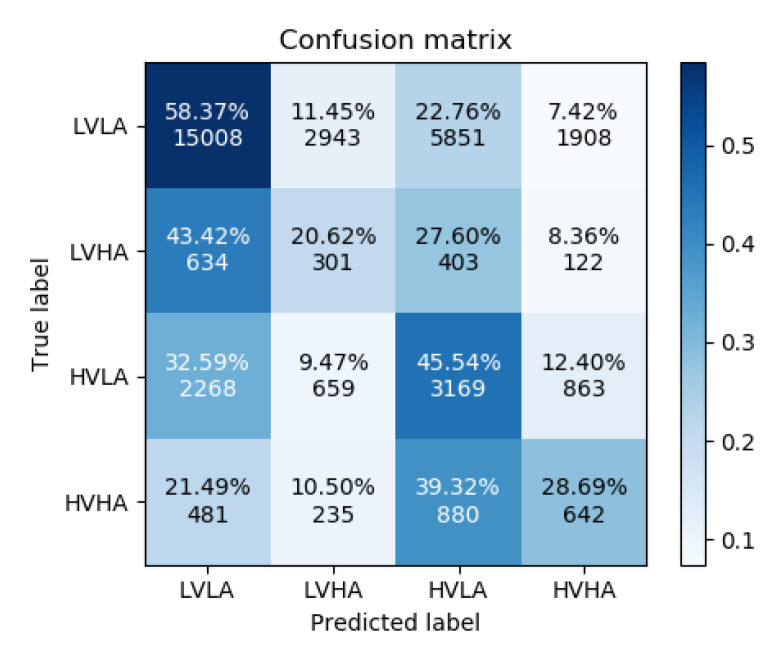
Confusion Matrix for Experiment 1.

**Figure 6:**
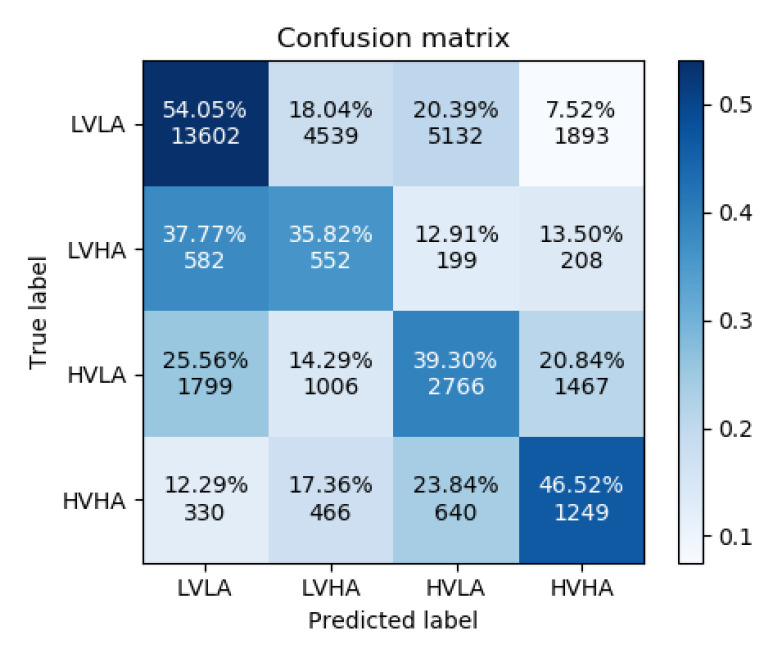
Confusion Matrix for Experiment 2.

Figure 5 and Figure 6 show the confusion matrices for experiments 1 and 2, respectively. As can be observed in their differences, the addition of the ECG and GSR bioelectrical signals yielded an increase in correctly classified high-arousal classes for both low and high valence. This is an increase of approximately 15 percentage points for the low valence samples and 18 percentage points for the high valence samples. High arousal classes were less often confused for low arousal classes; however, the misclassification of high arousal samples as their corresponding opposite valence state increased slightly.

A comparison of the accuracy results for valence and arousal with baseline methods can be found in Table 10. The works in question are: End-to-end facial and physiological model for Affective Computing and applications’ [77], An Attribute-invariant Variational Learning for Emotion Recognition Using Physiology [78], Using Deep Convolutional Neural Network for Emotion Detection on a Physiological Signals Dataset (AMIGOS) [80], and An inter-domain study for arousal recognition from physiological signals [79]. The comparison of the graph neural network for emotions was done with these baseline models that perform emotion classification using bioelectrical signals from the AMIGOS dataset.

The presented results show accuracy similar to the other baseline method, which allows GraphEmotionNet to be utilized in the analysis of the recognized emotion state vectors representing the six basic emotions and neutral state.

### 5.2. Determining the Method of Measuring Correctness of Recognized Emotions

Having a set of about 100 pairs of emotional state vectors annotated by five persons and the average value calculated, three classes of pairs were determined. In the class with consistent pairs (with the average value of annotations equal to 1.5), all pairs have a similarity measure greater than 0.8. In the class with the inconsistent pairs (with an annotation average value less than 0.5), about 40% of pairs have a similarity measure less than 0.5. There are no other classes where such pairs exist. Neither the consistent one nor the semi-consistent one contains pairs where the similarity measure is less than 0.5. The semi-consistent class (with an annotation average value in the range <0.5, 1.5) has the highest standard deviation for annotations (0.3 versus 0.19 and 0.14 for the consistent and inconsistent classes, respectively). This shows that this class is most subjective. These experimental premises were used to designate thresholds:

- consistent class-cosine similarity ≥ 0.8;
- semi-consistent class-cosine similarity in the range <0.5, 0.8);
- inconsistent class-cosine similarity < 0.5.

### 5.3. Analysis of Correctness of Recognized Emotional State Vectors

The analysis of the correctness of the recognized emotions is done using the cosine similarity measure and determined thresholds. It is worth remembering that the analysis does not compare the results with baseline methods as, to the best of our knowledge, DE features have only been used to recognize emotions in Circumplex models or for three emotions categories (positive, negative, and neutral). The presented analysis is mainly focused on the new finding connected with Ekman’s emotion recognition based on DE features and the GNN, which are discussed.

**Figure 7:**
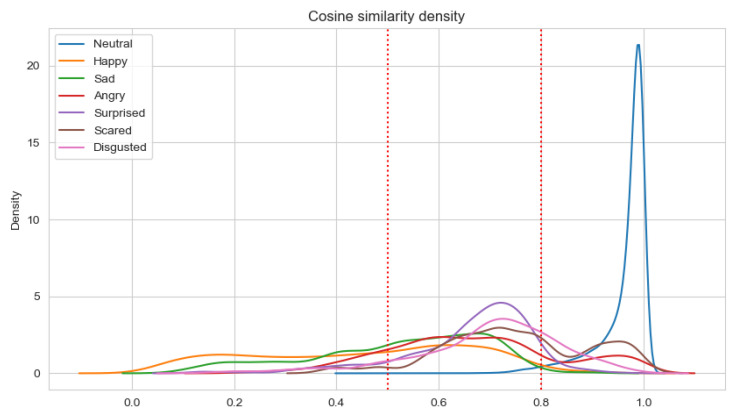
Density plot for Ekman’s emotions with neutral state in Experiment 3.

**Figure 8:**
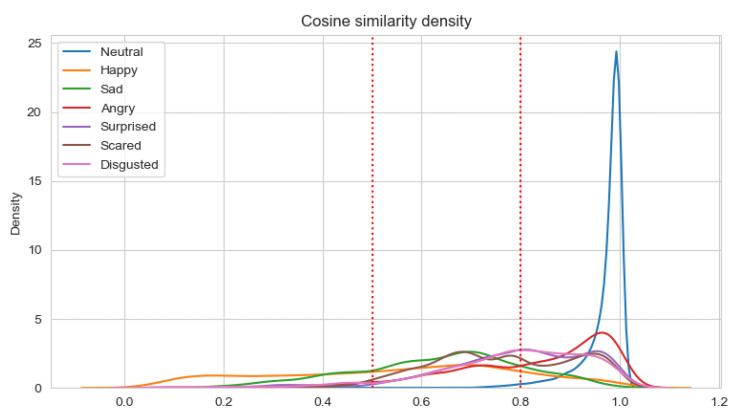
Density plot for Ekman’s emotions with neutral state in Experiment 4.

**Figure 9:**
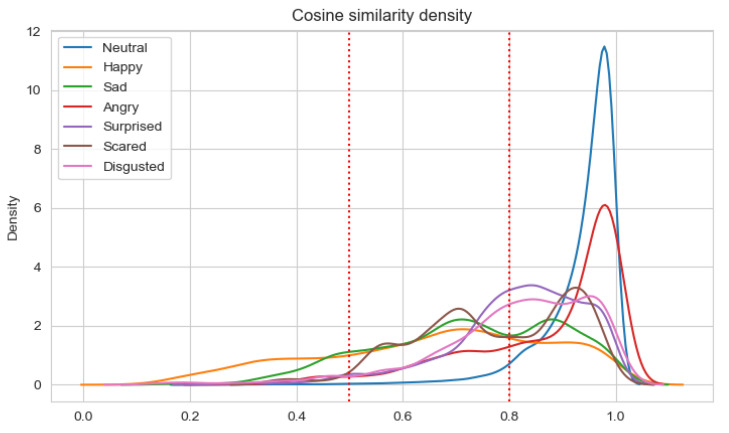
Density plot for Ekman’s emotions with neutral state in Experiment 5.

**Figure 10:**
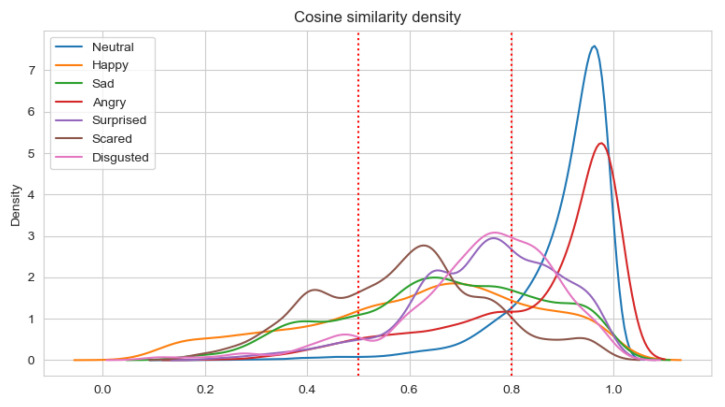
Density plot for Ekman’s emotions with neutral state in Experiment 6.

The results of the analysis are presented in Table 11 and Table 12 for Experiments 3–6, respectively. When the average cosine similarity is calculated for the specific emotion, those samples are taken into account for which the dominant emotion is the same. The percentages of consistent samples are depicted on density plots, in additional to Table 11 and Table 12. The density plots are prepared for a sliding window with a size 0.5 and are depicted in Figure 7, Figure 8, Figure 9 and Figure 10 for Experiments 3–6, respectively.

For Experiments 3 and 4, the average cosine similarity, as well as the percentage of consistent samples, is very high. This results from the fact that for about 91% of samples, the neutral emotional state is dominant. When the neutral state is dominant, the average cosine similarity is very high (96%/97% for the unimodal/multimodal approach). For the basic Ekman’s emotions, the average cosine similarity is the highest for fear and disgust when the unimodal approach is applied, and for anger and surprise in the multimodal approach. However, the average cosine similarities for fear and disgust are almost equal to that for surprise. Using the multimodal approach results in higher values of the cosine similarity. Adding new modalities influences the value of the average cosine similarity for anger the most. There is quite an interesting observation that the average cosine similarity is lowest for the happiness emotion.

Analyzing the percentage of consistent samples, it can be noticed that adding new modalities increase these percentages for all dominant emotions. The smallest difference in this percentage is for the fear emotion (analogically, the difference in the cosine similarity for fear is the lowest). Even more interesting is the fact that only the fear emotion has the highest percentage of consistent samples for the unimodal approach, which is not the case when the ECG and GSR observation channels are also analyzed. For the multimodal approach, the percentage is the highest for anger, but disgust and surprise also have higher percentages of consistent samples than fear. The cosine similarity threshold of 0.5 is exceeded by more than 80% of samples for anger, disgust, surprise, and fear in both the unimodal and multimodal approaches.

In the case of weighted classes, comparing the average cosine similarity for one modality used (Experiment 5) with those obtained in experiment 3, it can be noticed that the average cosine similarity increased for happiness, anger, surprise, and sadness. It is lower for the two highest obtained in Experiments 3 (i.e., disgust and fear). Comparing the average cosine similarity for the multimodal approach (Experiments 4 and 6), it is lower in Experiment 6 for all emotions but happiness and anger. Additionally, no significant change was observed to the cosine similarity values of the happiness, surprise, and sadness emotion classes when comparing the unimodal and multimodal approaches in Experiments 5 and 6. However, there was a difference in the cases of the remaining classes—anger, disgust, and fear. As can be seen in Table 12, both anger and disgust show marginal improvement, while the resulting cosine similarity for fear worsened. The percentage of non-consistent samples is lower for all emotions except for fear in the multimodal approach.

To summarize, the following observations were made:

- The cosine similarity for happiness and sadness is lower compared to the other basic emotions—this may be explained by the fact that the recognized facial expressions typical for sadness and happiness may have no reflection in a person’s emotions (e.g., the person can smile but she/he does not have to feel happy); there may be no correspondence between a person’s facial expressions and the emotions reflected in physiological signals;
- The best recognized emotion is anger, which may be a premise to formulate the conclusion that DE features convey much information about this emotion;
- Adding the ECG and GSR modalities in the process of emotion recognition increases the cosine similarity for almost all basic emotions except for fear. This may be explained by the fact that the emotion of fear is better reflected in EEG biosignals than in ECG or GSR; however, this result must be confirmed with additional research;
- Setting the class weights to increase the sensitivity of Ekman’s emotions increases the cosine similarity for happiness and sadness, but their cosine similarity is still the lowest of all of the basic emotions;
- Setting the class weights to increase the sensitivity influenced the percentage of consistent samples for the one modality (EEG) approach most—it increases this percentage significantly for happiness, anger, surprise, and sadness while in the same time reducing it by almost 30% for fear.

## 6. Discussion

This study presents an approach based on GraphSleepNet for emotion recognition. The GraphEmotionNet graph neural network was constructed by adapting GraphSleepNet. The answers to the research questions made it possible to validate the presented approach.

First, to answer RQ1 (What is the accuracy of valance and arousal predicted by GraphEmotionNet?), two experiments (1 and 2) were conducted. Within these experiments, the accuracies for both the unimodal and multimodal approaches were calculated. The results show accuracy similar to other baseline methods which permits GraphEmotionNet to be utilized for emotion recognition.

Second, to answer RQ2 (What is the correctness of the emotion state vectors for Ekman’s model with the neutral state predicted by GraphEmotionNet for a single moment of time?) and RQ3 (In what way do the modalities used in GraphEmotionNet and the emotion recognized influence the answers of RQ2?), three experiments (3–6) were conducted. Within these experiments, the cosine similarities of the obtained vectors were analyzed. Moreover, the thresholds allowing for the classification of samples as consistent, semi-consistent, and non-consistent were settled and the percentages of samples classified to these classes were calculated. Some observations were made in addition to the numerical metrics.

The study allowed us to answer the research questions RQ1, RQ2, and RQ3, and as a main contribution to the knowledge, formulate some observations about the recognition of the six basic emotions from the DE features. Still, the research has some limitations. The ground truth was obtained in the process of emotion recognition from facial expressions. This can, in some cases, be invalid as not all facial expressions must be reflected in the physiology. However, assuming that such cases can take place, the observations are still valuable as the accuracy of the emotion recognition from Face Reader for the AMIGOS dataset with respect to external annotations is 72% and 78% for valence and arousal, respectively. It is worth noting that the accuracy is calculated for the Circumplex model and not the Ekman one. What is more, the observations are currently identified based on the analysis of only one dataset. Taking the above into consideration, the observations must be treated as new detections and not general rules. To prove detections and define more general conclusions, the research should be repeated for at least two other datasets and it would be best for the ground truth to be obtained from various annotations (both self and external). Such an approach, however, demands a dataset to be annotated continuously in the Ekman model, and such datasets are currently not available.

Further to the obtained observations, the study also provides some knowledge about the similarity of emotional state vectors. It is difficult to set the thresholds unambiguously, even by carrying out experiments, as the perception of emotions is subjective, and the same feature influences the values of annotations describing the similarity of emotion state vectors.

The last significant achievement of the study is the validation of the GNN network for emotion recognition built for both the unimodal and multimodal approaches based on DE features.

The study also opens up new possibilities for further research. There are works planned to explore at least the following areas. It would be interesting to explore whether adding the contextual information about the obtained physiological signals (information characterizing their origin and meaning) can improve the correctness of GraphEmotionNet. Another approach that could be taken into consideration is the change of extracted features from the bioelectrical signals, using other features such as Power Spectral Density (PSD). It would also be worthwhile to check the correctness from a different perspective, that is, the correctness of the whole time series representing the emotional states during the whole activity (not only in a single moment of time). To sum up, the presented research provides some new insights, especially in the context of Ekman’s emotion recognition from physiological signals.

## Figures and Tables

**Table 1:** Recognized emotions from datasets.

Article	Recognized Emotion	Predicted Label	Dataset
[41]	positive, neutral, negative	single label	SEED
[14]	Ekman model (6 basic emotions)	single label	DEED, MPED
[42]	positive, neutral, negative	single label	SEED, RCLS
[15]	valence and arousal	multiple labels	DEAP, ASCERTAIN
[16]	valence and arousal	multiple labels	AMIGOS

**Table 2:** Basic description of five multimodal affective datasets.

Name	Type of Signal	Number of Participants	Type of Videos Watched
AMIGOS	EEG (14 channels), ECG (2 channels), GSR (1 channel)	40	16 movie clips with lengths between 50 and 150 s
DEAP	EEG (32 channels), ECG (2 channels), GSR, EOG	32	40 one-minute long music videos
ASCERTAIN	EEG, ECG, GSR, facial features	58	36 movie clips with lengths between 51 and 127 s
DREAMER	EEG (14 channels), ECG (2 channels)	23	18 movie clips with lengths between 65 and 393 s
SEED	EEG (62 channels)	15	15 movie clips (duration of every video approx. 4 min)

**Table 3:** Hyperparameters in the model that predicts emotions to Ekman’s model with additional neutral emotion.

Hyperparameter Description	Value
Layer number of ST-GCN	1
Standard convolution kernels	10
Graph convolution kernels	10
Chebyshev polynomial K	3
Regularization parameter	0.001
Dropout probability	0.4
Batch size	2048
Learning rate	0.001
Optimizer	Adam

**Table 4:** Hyperparameters in the model that predicts emotions to Circumplex model.

Hyperparameter Description	Value
Layer number of ST-GCN	1
Standard convolution kernels	10
Graph convolution kernels	10
Chebyshev polynomial K	3
Regularization parameter	0.001
Dropout probability	0.5
Batch size	512
Learning rate	0.001
Optimizer	Adam

**Table 5:** Matrix row representing emotions detected by Face Reader for the first participant watching the 80th video.

Part.	Movie	Neutral	Disgust	Happiness	Surprise	Anger	Fear	Sadness
...	...	...	...	...	...	...	...	...
1	80	1	1	1	1	0	0	0
...	...	...	...	...	...	...	...	...

**Table 6:** Matrix row representing annotated emotions for the first participant watching the 80th video.

Part.	Movie	Neutral	Disgust	Happiness	Surprise	Anger	Fear	Sadness
...	...	...	...	...	...	...	...	...
1	80	0	0	0	0	1	0	0
...	...	...	...	...	...	...	...	...

**Table 7:** Example showing the method of determining internal consistency of self-annotations.

Participant	1
Movie	19
Annotated Valence	1.00
Annotated Arousal	6.46
Quadrant	LVHA
Possible Ekman emotions	Disgust, Anger, Fear
Annotated Ekman emotions	Disgust, Fear
Consistent	Yes

**Table 8:** Experiments specification for analysis of accuracy of recognized quadrant in Circumplex model.

Exp. ID	Emotion Model	Channels	Data	Network Parameters
Exp. 1	Circumplex model	EEG	Data annotated with valence and arousal	Table 4
Exp. 2		EEG, ECG, GSR		

**Table 9:** Specification of experiments.

Exp. ID	Emotion Model	Channels	Data	Network Parameters
Exp. 3	Ekman model with neutral emotion	EEG	Data annotated with emotions obtained from facial expression analysis	Table 3
Exp. 4		EEG, ECG, GSR		
Exp. 5	Ekman model with neutral emotion	EEG	Data annotated with emotions obtained from facial expression analysis	Table 3 and also class weights that increase sensitivity of Ekman’s emotions
Exp. 6		EEG, ECG, GSR		

**Table 10:** Comparison of accuracy found in the literature.

	Method	Valence Acc	Arousal Acc
[77]	AE	65.05%	87.53%
[78]	AI-VAE	68.80%	67.00%
[79]	DCNN	76.00%	75.00%
[80]	AdaB	-	56.00%
Our method (unimodal)	GNN	67.20%	75.88%
Our method (multimoadl)	GNN	69.71%	70.75%

**Table 11:** Analysis of cosine similarity for Experiments 3 and 4.

	Average Cosine Similarity		Percentage of Consistent Samples		Percentage of Semi-Consistent Samples		Percentage of Non-Conistent Samples	
	**EEG**	**EEG, ECG, GSR**	**EEG**	**EEG, ECG, GSR**	**EEG**	**EEG, ECG, GSR**	**EEG**	**EEG, ECG, GSR**
Happiness	0.4570	0.5499	02.13%	16.32%	45.77%	44.15%	52.09%	39.53%
Anger	0.6663	0.8246	19.49%	63.34%	64.31%	32.57%	16.21%	04.09%
Disgust	0.6950	0.7755	21.87%	50.83%	68.09%	42.41%	10.04%	06.77%
Surprise	0.6625	0.7893	05.94%	53.00%	83.33%	42.72%	10.72%	04.28%
Sadness	0.5251	0.6369	00.82%	16.49%	61.58%	62.70%	37.60%	20.81%
Fear	0.7527	0.7676	34.26%	38.68%	60.19%	59.43%	05.56%	01.89%
Neutral	0.9635	0.9707	98.07%	98.59%	01.92%	01.37%	00.01%	00.04%
All	0.9270	0.9457	89.29%	93.00%	08.12%	05.41%	02.59%	01.59%

**Table 12:** Analysis of cosine similarity for Experiments 5 and 6.

	Average Cosine Similarity		Percentage of Consistent Samples		Percentage of Semi-Consistent Samples		Percentage of Non-Conistent Samples	
	**EEG**	**EEG, ECG, GSR**	**EEG**	**EEG, ECG, GSR**	**EEG**	**EEG, ECG, GSR**	**EEG**	**EEG, ECG, GSR**
Happiness	0.6076	0.6084	22.71%	21.66%	43.54%	47.34%	33.75%	31.00%
Anger	0.8069	0.8357	59.07%	66.69%	33.78%	26.72%	07.15%	06.59%
Disgust	0.6824	0.7255	19.68%	34.70%	68.96%	55.15%	11.36%	10.16%
Surprise	0.7211	0.7207	32.67%	33.19%	57.95%	60.31%	09.38%	06.50%
Sadness	0.6147	0.6178	17.77%	18.01%	56.23%	58.60%	25.99%	23.39%
Fear	0.6158	0.5743	06.48%	08.33%	75.00%	63.89%	18.52%	27.78%
Neutral	0.9088	0.9091	90.52%	89.19%	08.68%	10.09%	00.80%	00.72%
All	0.8868	0.8880	84.53%	83.72%	13.00%	13.99%	02.47%	02.29%

## Data Availability

Data was obtained from Queen Mary University of London, London, UK & University of Trento, Trento, Italy and are available at http://www.eecs.qmul.ac.uk/mmv/datasets/amigos/ (accessed on 5 April 2022) with the permission of Queen Mary University of London, London, UK.
